# Safety Evaluation of an Intranasally Applied Cocktail of *Lactococcus lactis* Strains in Pigs

**DOI:** 10.3390/ani13223442

**Published:** 2023-11-08

**Authors:** Ruth Rattigan, Lukasz Wajda, Abel A. Vlasblom, Alan Wolfe, Aldert L. Zomer, Birgitta Duim, Jaap A. Wagenaar, Peadar G. Lawlor

**Affiliations:** 1 Teagasc Pig Development Department, Animal and Grassland Research and Innovation Centre, Moorepark, Fermoy, P61 C996 County Cork, Ireland; 2 Faculty of Veterinary Medicine, Utrecht University, Yalelaan 1, 3584 CL Utrecht, The Netherlands; 3 School of Veterinary Medicine, University College Dublin, Belfield, D04 V1W8 Dublin, Ireland

**Keywords:** pigs, LA-MRSA, bacterial cocktail, safety, growth, histopathology, gene expression

## Abstract

**Simple Summary:**

In this study, the safety of an intranasally applied cocktail of *Lactococcus lactis* was evaluated in new-born pigs. Prior to farrowing, twelve sows were assigned to either a placebo or cocktail group. Immediately after farrowing, either the placebo or cocktail was applied to both nostrils of all piglets in the corresponding group. Piglet health and growth were monitored at regular intervals, and on three occasions piglets were necropsied and samples were collected from the respiratory tract to identify any changes caused by the treatment. The results show that the cocktail did not negatively impact piglet growth or health, nor did it cause histological changes in the nasal conchae, tonsils or lung tissues; but it did alter the gene expression of *pBD2*, *TLR9* and *IL-1β* in the nasal conchae. These findings indicate the cocktail is safe for administration to pigs.

**Abstract:**

Three *Lactococcus lactis* strains from the nasal microbiota of healthy pigs were identified as candidates for reducing MRSA in pigs. The safety of nasal administration of a cocktail of these strains was examined in new-born piglets. Six days pre-farrowing, twelve sows were assigned to the placebo or cocktail group (*n* = 6/group). After farrowing, piglets were administered with either 0.5 mL of the placebo or the cocktail to each nostril. Health status and body weight were monitored at regular time points. Two piglets from three sows/treatment group were euthanised at 24 h, 96 h and 14 d after birth, and conchae, lung and tonsil samples were collected for histopathological and gene expression analysis. Health scores were improved in the cocktail group between d1–5. Body weight and daily gains did not differ between groups. Both groups displayed histological indications of euthanasia and inflammation in the lungs, signifying the findings were not treatment related. The expression of *pBD2*, *TLR9* and *IL-1β* in the nasal conchae differed between groups, indicating the cocktail has the potential to modulate immune responses. In summary, the *L. lactis* cocktail was well tolerated by piglets and there was no negative impact on health scores, growth or lung histopathology indicating that it is safe for administration to new-born piglets.

## 1. Introduction

*Staphylococcus aureus* is a gram-positive bacterium commonly identified on the skin and mucosa, with the nasal cavity being one of the most frequently colonised regions in humans and animals [1,2]. *S. aureus* is a facultative pathogen, with methicillin-resistant *S. aureus* (MRSA) being the leading bacterial cause of several diseases including skin, bone and joint infections, endocarditis, bacteraemia and other hospital-acquired infections [3,4] in humans. Livestock-associated MRSA (LA-MRSA) is a zoonotic pathogen that colonises pigs and other livestock species [2,5,6]. There appears to be an age-related susceptibility to *S. aureus* in pigs, with colonisation after 10 weeks of age being much less frequent than in younger pigs, and sows are thought to be the primary source of LA-MRSA transmission to piglets pre-weaning [7]. While LA-MRSA rarely causes clinical infections in pigs, it is a public health concern as it can be transmitted from pigs to humans [2,8,9]. Transmission may occur through direct contact with infected pigs and dust, or through contamination of the environment, feed and flooring/pen materials [10,11]. Dust particles carrying LA-MRSA could be transferred via clothing or equipment to other environments including farms and homes, therefore enabling transfer to animals/humans outside of the source farm. Humans that are in close contact with animals/pigs have a higher risk of being colonised with LA-MRSA than the general population, this includes farm workers, abattoir workers and veterinarians [12,13].

While LA-MRSA is not currently a major public health concern due to low incidence in humans [2], it can manifest with similar clinical presentations as hospital or community acquired MRSA, with skin and soft tissue, ear, eye, respiratory, bone and joint and blood infections reported [14,15]. Furthermore, it is possible that LA-MRSA strains will acquire additional virulence factors and resistance genes which may lead to the emergence of more virulent strains [13]. Therefore, strategies to reduce or eliminate LA-MRSA on farms are receiving attention. One strategy is the implementation of a strict surveillance program, which involves testing pigs, pig farm workers and their families, and the depopulation of MRSA positive farms. This system is applied in Norway and appears to be effective given the low prevalence of MRSA in Norwegian pigs; however, it is an expensive and time-consuming strategy [12,16]. Another option is the nasal application of probiotic bacteria which are negatively associated with *S. aureus*/MRSA abundance, and therefore could potentially inhibit their growth [17]. Probiotic bacteria can protect the host from pathogenic bacteria through competitive exclusion and/or the production of antimicrobial compounds or stimulation of the immune system [18,19].

Work undertaken by Vlasblom et al. (manuscript in preparation) identified three strains of *Lactococcus lactis* from nasal swabs collected from healthy piglets, which were negatively correlated with MRSA using metagenomics and qPCR data. In addition to the negative correlations with MRSA, the strains were chosen based on the results of in vitro (alpha and beta haemolytic activity and phenotypic antimicrobial resistance) and in silico (whole genome sequencing, taxonomy, antimicrobial resistance genes and virulence factors) safety testing in accordance with the European Food Safety Authority (EFSA) guidance. As the nasal application of probiotics is quite novel, with only few studies to date [20,21,22], it was necessary to ensure the safety of application prior to investigating the efficacy of the probiotic mixture at a larger scale. Thus, the objective of this experiment was to assess the safety of intranasal administration of this consortium of *L. lactis* strains in new-born pigs. The safety of the bacterial cocktail was evaluated by monitoring piglet health and body weight, with histological assessment of the nasal conchae, tonsils and lungs, and analysis of the expression of immune markers in the nasal conchae.

## 2. Materials and Methods

### 2.1. Preparation of Bacterial Cocktail

The bacterial cocktail consisted of three *Lactococcus lactis* strains: EW01, EW04 and EW05 strains, which were selected based on their negative correlations with *S. aureus* and MRSA via metagenomic analysis of nasal swabs collected from pigs in an earlier study. The strains also successfully completed in vitro and in silico safety testing (i.e., alpha and beta haemolytic activity, phenotypic antimicrobial resistance testing, whole genome sequencing and subsequent analysis including taxonomy, and searches for antimicrobial resistance genes and virulence factors) before being applied in vivo. The bacterial cocktail was prepared by combining cell stocks (suspended in 20% glycerol (Sigma Aldrich, Saint Louis, MO, USA)) of the *L. lactis* strains. Stocks were stored at −80 °C and thawed at 4 °C 72 h before the expected farrowing date. The concentration of each stock was determined by performing a serial dilution with a maximum recovery diluent (MRD, Merck, Darmstadt, Germany) and plating on De Man, Rogosa and Sharpe (MRS) agar (Merck). The colonies were counted following incubation at 37 °C for 72 h. The appropriate volumes of each stock were pooled, and the volume was made up to 100 mL with sterile phosphate buffered saline (PBS, pH 7.2–7.6, Sigma Aldrich) so that the final concentration of each strain was 3.33 × 10^8^ CFU/mL (1 × 10^9^ CFU/mL in total). For the placebo, the same volume of sterile 20% glycerol solution in PBS was used. Sterile 1.5 mL graduated Pasteur pipettes were prefilled with 0.55 mL (0.05 mL extra to allow for losses during administration) of either the placebo or the bacterial cocktail, reversed, put on the racks and then transferred to two separate cool boxes filled with ice until administration.

### 2.2. Experimental Design and Animal Management

Twelve healthy pregnant sows (Large White × Landrace) which had not received antimicrobials in the previous 30 days were selected based on their expected farrowing date, blocked by parity and assigned to one of two groups, the placebo or cocktail group (*n* = 6 sows/group). Six days prior to their expected farrowing date (d-6), the sows were transferred to one of two farrowing rooms. Prior to the housing of the sows, each farrowing room was cleaned, disinfected and allowed sufficient time to dry according to common practice in the facility. At ~day 110 of gestation, sows were moved into standard farrowing crates in pens (dimension: 2.5 m × 1.8 m) with cast-iron slats under the sow and plastic slats with a water heated floor pad for the piglets (BigDutchman; Vechta, Germany). Farrowing room temperature was maintained at ~24 °C at the time of farrowing and gradually reduced to 21 °C by d7 of lactation. The temperature of the heat pads was 38–40 °C for the first 2 days after farrowing and was reduced by 1 °C each day to 30 °C at 10 days after farrowing and it was maintained at this temperature until weaning. Artificial lighting was provided daily from 08:00 h to 16:30 h. During the experimental period, sows in both groups were fed a standard gestation/lactation diet (Supplementary Table S1). Twenty-four hours before their expected farrowing date, sows were induced with 2 mL of the synthetic prostaglandin analogue Planate (0.0875 mg Cloprostenol as 0.092 mg Cloprostenol Sodium per ml; Intervet Ireland Ltd. Magna Drive, Magna Business Park, Citywest Road, Dublin, Ireland) via deep intramuscular injection. Sows farrowed over a 5-day period and cross-fostering of piglets was performed between 24 and 48 h postpartum to equalize litter size, but only within the same group. Pigs that were cross-fostered were not used for sampling purposes. The average litter sizes were 16 piglets/litter (96 in total) and 18 piglets/litter (108 in total) for the placebo and cocktail groups, respectively.

Within 24 h of birth, piglets were individually weighed and tagged, and their teeth were clipped. Following this, all piglets in the placebo and cocktail groups were administered either the placebo or the cocktail, respectively. For the placebo group, 0.5 mL of sterile PBS containing sterile 20% glycerol was applied to each nostril of all piglets (1 mL in total per pig). Subsequently, all piglets in the bacterial cocktail group were administered 0.5 mL of sterile PBS containing a multi-strain cocktail of three *Lactococcus lactis* strains in equal concentrations (3.33 × 10^8^ CFU/mL) to each nostril (1 mL in total per pig). The intranasal delivery of the placebo or cocktail treatments was applied 1 cm into the anterior nares after cleaning of the external nasal area with a dry Kimcare medical wipe (Galleon Supplies Ltd., Coventry, West Midlands, UK). Piglets requiring antimicrobial treatment in the farrowing rooms were recorded, moved to a separate pen within each room and not subsequently sampled.

At 24 h, 96 h and 14 d after birth, six piglets from both treatment groups were selected for necropsies and sample collection (2 piglets of median birth weight/time point from each of 3 sows per treatment). The piglets remaining after the necropsies were weaned on the same date (24, 27 or 28 days of age) and moved into two separate weaner rooms (one for the placebo group and one for the bacterial cocktail group). Post-weaning (PW), there were 78 pigs remaining in the bacterial cocktail group (~13 pigs/litter) and 67 pigs in the placebo group (11.2 pigs/litter). Piglets remained within their litter group PW, with each litter group moved to their own pen within the weaner accommodation where they remained until the end of the experiment (d74). In the weaner rooms, pens (2.5 m × 2.0 m) were fully slatted with plastic flooring (BigDutchman; Vechta, Germany). The temperature of the weaner rooms was maintained at 28 °C for the first 7 days PW, gradually reduced to 22 °C by d28 PW and maintained at 22 °C until the end of the experiment. Ventilation was from a punched ceiling with air exhausted via a variable speed fan linked to a thermostat that was controlled with a computer (Big Dutchman 135, BigDutchman; Vechta, Germany).

### 2.3. Diet Preparation and Feeding

Diets were formulated to meet or exceed National Research Council (NRC) recommendations [23]. The ingredient composition and nutrient content of the diets are shown in Supplementary Table S1. During gestation, sows were fed a gestation diet (Diet 1) in meal form. The gestation diet was formulated to have a total lysine content of 0.8% and an energy density of 12.5 MJ DE/kg. In the farrowing room, diets were fed using a computerized feed delivery system (DryExact Pro, Big Dutchman, Vechta, Germany). Sows were fed a lactation diet (Diet 2; Supplementary Table S1) in meal form twice daily from farrowing to day 6 of lactation and three times daily from day 7 to weaning. Sows were fed according to a lactation feeding curve which started at 60 MJ DE/day at day 0 of lactation and gradually increased to 107, 125, 133 and 137 MJ DE/day at days 7, 14, 21 and 26 of lactation, respectively. During lactation, feeding curves for individual sows were adjusted up or down, as required, to ensure that sow feed intake was as close to ad libitum feeding as possible and to prevent feed wastage. Water was provided on an ad libitum basis to sows from a single-bite drinker in the feed trough and to suckling piglets from a bowl in the farrowing pen. The starter diet (Diet 3; Supplementary Table S1) was fed in pelleted form (3 mm diameter pellets) as creep feed to suckling pigs from day 11 after birth until weaning using a creep feeder (Easy pan; Rotecna, Lleida, Spain) placed at the bottom of the heat pad.

Following weaning, pigs were fed a sequence of diets in accordance with their growth stage. The starter diet (Diet 3; Supplementary Table S1) was provided from weaning to day 6 PW, the link diet (Diet 4; Supplementary Table S1) from days 6 to 17 PW and the weaner diet (Diet 5; Supplementary Table S1) from day 17 PW to the end of the experiment. All diets fed PW were in pelleted form (3 mm diameter), and all were provided on an ad libitum basis. Diets were fed using single-space (33 cm) wet-dry feeders (MA19100, Verba, The Netherlands) with inset nipple drinker and a supplementary bowl drinker (SS Drinker, Rotecna, Spain).

### 2.4. Data Recording, Sampling and Analysis

#### 2.4.1. Litter Data at Birth and Piglet Growth Performance and Mortality

Reproductive parameters were recorded per litter at birth; that is, the number of piglets (total born, born alive and stillborn) and the weight and sex of each piglet, and each piglet was tagged for identification purposes. Thereafter, piglets were individually weighed at weaning and at the end of this study (74 days of age) and these data were used to determine pre- and post-weaning average daily gain (ADG). Piglet mortality between birth and weaning was also recorded.

#### 2.4.2. Safety Evaluation—Health Scores and Necropsies

Three litters per treatment were selected for daily health scoring and for necropsy and sample collection. Health checks were completed daily in the farrowing rooms, and on days 46, 61 and 74 in the weaner rooms. These checks included faecal consistency scoring, and observations for normal behaviour, dehydration and clinical signs of illness as detailed in Supplementary Table S2. The threshold for the administration of treatment was a score of three in a single category or an accumulated score of eight. Any animals that needed antimicrobial treatment were removed from the trial. All veterinary treatments were recorded, including the identity of the pig, the symptoms, the medication used and the dosage. A portion of the animals that died during this study were taken to the Regional Veterinary Laboratory (Cork, Ireland) for full postmortem examination. As outlined above, at 24 h, 96 h and 14 days after birth, 6 piglets from each treatment group were euthanised with lethal injection with pentobarbital sodium (Dolethal 200 mg/mL; Vetoquinol Ireland Limited, 12 Northbrook Road, Ranelagh, Dublin 6, Ireland) in a room separate from other pigs. Subsequently, the following samples were collected for histopathological analysis and RNA extraction: conchae, left and right cranial, medial and caudal lungs, left and right palatine tonsils, left and right pharyngeal tonsils and left and right paraepiglottal tonsils. Samples for histopathological analysis were fixed in 10% neutral-buffered formalin (Lennox Laboratory Supplies, Bluebell, Dublin, Ireland) and stored at room temperature before processing. Samples for the RNA extraction were suspended in RNAlater (Sigma Aldrich, Saint Louis, MO, USA) overnight, before removing the RNAlater and storing at −80 °C.

#### 2.4.3. Histopathology Analysis

For histological analysis, tissues were routinely processed for paraffin embedding. Tissues were cut into 3 μm thick sections and stained with hematoxylin and eosin (H&E). All tissue slides were examined by the same pathologist and scored using subjective criteria. Pathological changes observed included presence of inflammatory cells, alveolar oedema fluid, alveolar septal thickening, pulmonary interlobular oedema, collapse of alveolar spaces. Any changes seen were recorded under the grouping of mild, moderate or marked. No examples of severe changes were seen.

#### 2.4.4. RNA Extraction and Gene Expression Analysis

Total RNA was isolated from the nasal conchae; and as a control, peripheral blood mononuclear cells (PMBCs) stimulated with Concanavalin A (Sigma Aldrich, Saint Louis, MO, USA) were used. The PMBCs were previously harvested from pig spleen, isolated using a density gradient and intact cells were counted. Thirty mg of nasal conchae tissue and 10^6^ PBMCs were used for RNA isolation using the RNeasy kit (Qiagen Benelux, Venlo, The Netherlands) according to the manufacturer’s instructions with some minor modifications. For both sample types, the disruption and homogenization step was performed using Lysing Matrix D bead tubes (MP Biomedicals) and the Tissuelyser LT in buffer RLT (Qiagen Benelux). After eluting the RNA, the eluate was treated with DNase 1 (ThermoFisher Scientific, Eindhoven, The Netherlands) to remove DNA according to the manufacturer’s instructions.

Total RNA was reverse transcribed using SuperScript™ IV Reverse Transcriptase (Invitrogen, ThermoFisher Scientific, Waltham, MA, USA), using random hexamer primers in a final reaction volume of 40 μL according to the manufacturers protocol.

A total of 1 μL diluted cDNA was used for RT-qPCR analysis, which was performed with a Lightcycler 480 (Roche Diagnostics, Almere, The Netherlands) using SYBR-Green qPCR Master Mix (BIO-RAD, Veenendaal, The Netherlands) according to the manufacturer’s protocol. The thermocycling protocol was 95 °C for 30 s, followed by 45 cycles at 95 °C for 5 s and 60 °C for 31 s. The data were normalized to the β-actin housekeeping gene to account for repeated measures. The primers used to target the porcine *TNFα*, *CXCL8*, *IL1β*, *IL6*, *pBD2*, *TLR2* and *TLR9* were taken from Yang, et al. [24] and are listed in Supplementary Table S3. PCR products were analysed using a melting curve analysis and the fold changes were calculated using the 2^−ΔΔ^Cq method [25].

#### 2.4.5. Statistical Analysis

All data on health scores, growth performance, histology and gene expression were checked for normality using the univariate procedure in SAS^®^ 9.4 (SAS Institute Inc., Cary, NC, USA). Health score data were analysed using the GLIMMIX procedure of SAS^®^ 9.4 using a Gaussian distribution. The model included the effects of treatment and day and the associated interaction, with the pig being the experimental unit. As all piglets had a health score of zero after day 5, the scores from day 6 onwards were excluded from statistical analysis. As the histology data were in binary form (yes/no), the log of the odds was modelled using logistic regression using the GENMOD procedure in SAS. Treatment was included as a fixed effect in the model. Across all binary variables, odds ratios were calculated as the exponent of the model solutions. The growth performance data (live weight (LW), ADG and creep feed intake) were analysed using the MIXED procedure in SAS. For pre-weaning LW and ADG, treatment was included as a fixed effect and weaning age and initial body weight were included as covariates. For creep feed intake, treatment was included as a fixed effect in the model. For PW performance, treatment was included as a fixed effect and weaning weight as a covariate in the model. The experimental unit for all parameters was the pig within litter. The gene expression data were also analysed using the MIXED procedure in SAS. Treatment was included as a fixed effect, with the pig being the experimental unit. The results are presented as least square means with their standard errors. *p*-values were corrected for multiple comparisons using the Tukey adjustment. Differences between treatments were considered significant for *p* ≤ 0.05, while 0.05 < *p* ≤ 0.10 was considered a tendency.

## 3. Results

### 3.1. Health Scores

In the first five days, there were treatment, time and treatment × time effects on accumulated health scores (Table 1, *p* < 0.001). Overall, the cocktail treated piglets had a lower (improved) health score over the first five days compared to the placebo group (0.70 ± 0.046 vs. 1.38 ± 0.043). There was also an effect of time on health score, with scores reducing between days 1 and 5 (*p* < 0.0001). There was also a treatment × time interaction, whereby pigs in the cocktail group had lower (improved) health scores compared with the placebo group on days 2, 3 and 5 (*p* < 0.001). However, it is important to note that the accumulated scores were well below the threshold that would have required treatment for both groups, indicating there were no significant health concerns for either treatment group for the duration of the experiment. From day 6 onwards, all piglets in both treatment groups had accumulated health scores of 0. 

### 3.2. Litter Data and Pre- and Post-Weaning Growth

As described above, there were 96 (16 pigs/litter) piglets born alive to sows in the placebo group and 108 (18 pigs/litter) piglets born alive to sows in the bacterial cocktail group. Birth weight did not differ between the placebo and cocktail groups (1.53 ± 0.093 kg and 1.73 ± 0.065 kg, respectively; *p* = 0.120). Ten piglets died from the placebo group and twelve from bacterial cocktail group prior to weaning. In addition, two pigs from the placebo group and one pig from the cocktail group died PW. Of the 25 pigs that died or were euthanised throughout this study, 11 were sent for postmortem examination, 6 from the cocktail group and 5 from the placebo group. The causes of death identified during postmortem were crushing (one placebo), hypoglycaemia/starvation (one placebo and one cocktail), possible septicaemia due to *Escherichia coli* or *Streptococcus dysgalactiae* (three cocktail) and not determined (three placebo and two cocktail). Cause of death for the remaining pigs was identified by farm staff as poor viability (one placebo, five cocktail), crushing by sow (four placebo, one cocktail) and injuries/dislocations (two placebo, one cocktail). In addition, 11 piglets in the placebo group and 6 in the bacterial cocktail group were administered with antimicrobials prior to weaning and removed from this study. Reasons for antimicrobial administration included lethargy, lameness and infections.

The effect of treatment on growth performance is presented in Table 2. Treatment had no effect on weaning weight, ADG or total creep feed intake pre-weaning (*p* > 0.05). Similarly, PW, there was no difference in final body weight or ADG between the placebo and cocktail groups (*p* > 0.05).

### 3.3. Histopathology

**Figure 1:**
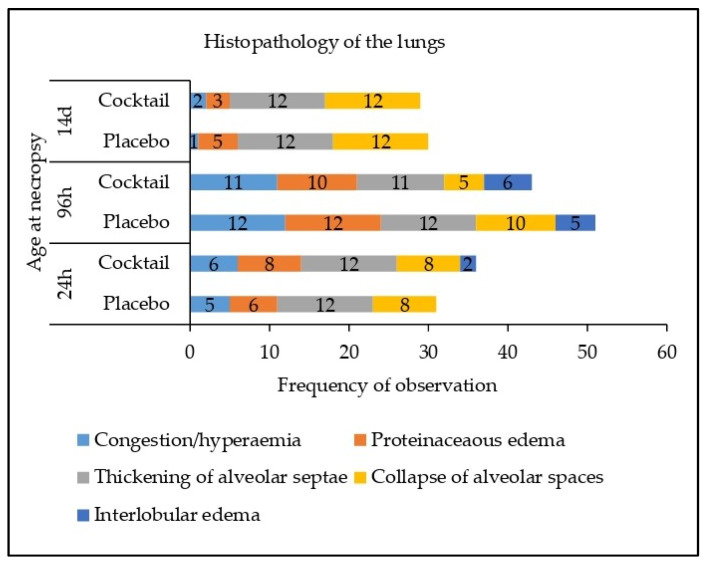
Effect of treatment on histopathology in the left lung and the right lung.

No significant abnormalities were identified in the conchae, palatine tonsils, paraepiglottal tonsils or retropharyngeal tonsils in either the placebo or cocktail treated groups (Supplementary Tables S4–S7). In the lungs, a number of abnormalities were identified in samples across both treatment groups. These included the observation of diffuse moderate congestion/hyperaemia, diffuse flooding with proteinaceous oedema and collapse of alveolar spaces, which are often associated with euthanasia. Other abnormalities included mild to moderate thickening of the alveolar septae with neutrophils and lymphocytes and mild to moderate interlobular oedema, these changes are indicative of an inflammatory process. These findings are summarised in Figure 1. There was no difference in the frequency of abnormalities between the two treatment groups at any time-point (24 h, 96 h or 14 d; *p* > 0.05).

### 3.4. Gene Expression in the Nasal Conchae

**Figure 2:**
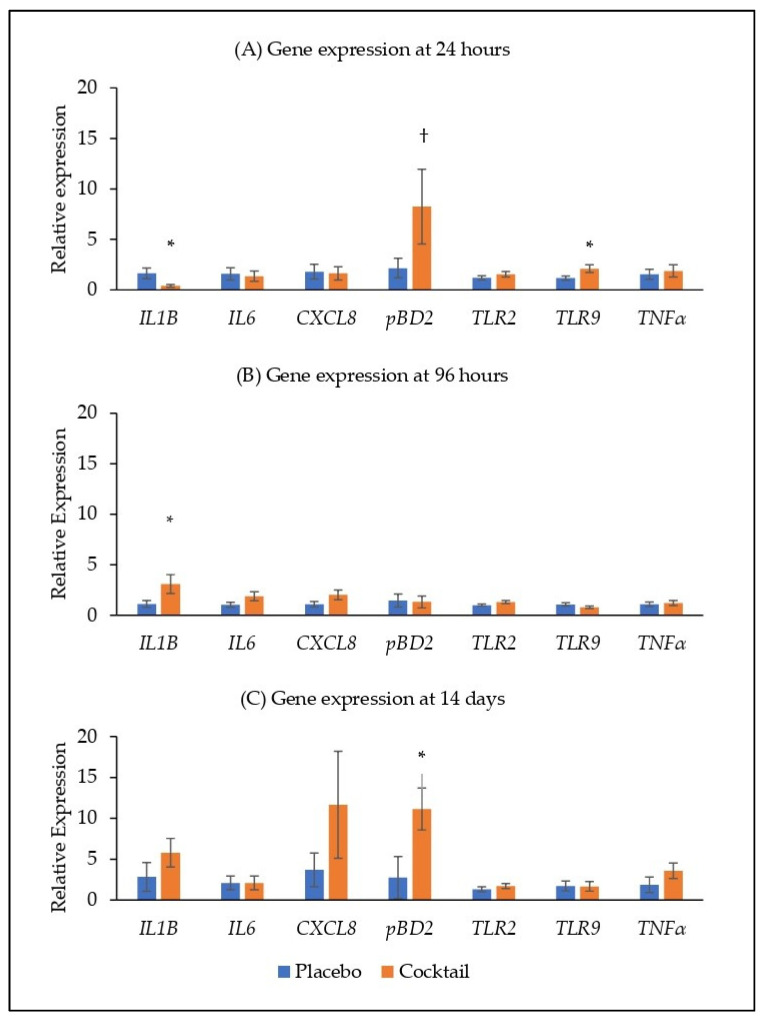
Relative gene expression in the nasal conchae at (**A**) 24 h, (**B**) 96 h and (**C**) 14 days post placebo/cocktail administration. *IL1β*, interleukin 1 beta; *IL6*, interleukin 6; *CXCL8*, C-X-C motif chemokine ligand 8/interleukin 8; *pBD2*, porcine beta defensin 2; *TLR2*, toll-like receptor 2; *TLR9*, toll-like receptor 9; *TNFα*, tumour necrosis factor alpha. Significant differences between treatments are indicated as * (*p* < 0.05) and † (*p* < 0.10).

At 24 h after administration, there was no difference in the expression of *IL6*, *CXCL8*, *TLR2* and *TNFα* in the nasal conchae (Figure 2A). The expression of *IL1B* was reduced in the bacterial cocktail group compared with the placebo group (*p* < 0.05), while the expression of *pBD2* and *TLR9* were higher in the bacterial cocktail group compared with the placebo group (*p* < 0.10 and *p* < 0.05, respectively; Figure 2A). At 96 h post administration, the expression of *IL1B* was higher in the bacterial cocktail group compared with the placebo group (*p* < 0.05; Figure 2B). At 14 d post administration, the expression of *pBD2* was higher in the bacterial cocktail group compared to the placebo group (*p* < 0.05), but there were no significant differences in the expression of the other target genes (Figure 2C).

## 4. Discussion

While the safety and efficacy of orally administered probiotics has been widely demonstrated in pigs [19], to date, there are fewer studies examining the administration of probiotics to the upper respiratory tract (URT) of humans and animals [20]. Although the species *L. lactis* has EFSA qualified presumption of safety (QPS) status, it is still necessary to demonstrate the in vivo safety of the cocktail of strains using this administration route. The health score, growth performance, histopathology and gene expression data demonstrate that the nasal administration of this probiotic cocktail is safe and well tolerated in new-born piglets. While this is the first example of administration of *L. lactis* to the nasal cavity of pigs, this species has previously been administered to the nasal cavity of human patients with chronic rhinosinusitis without adverse effects [21]. These data add to existing evidence on the safety of intranasal probiotic application in animal and human models for the prevention or treatment of respiratory diseases including influenza virus, pneumoviruses and bacterial respiratory infections [22].

The accumulated health scores show that the intranasal application of the bacterial cocktail did not adversely affect piglet health. After five days, piglets in both groups had accumulated health scores of zero, indicating that no health abnormalities were detected. Likewise, nasal administration of the *L. lactis* strains did not negatively impact pig growth performance. In contrast to these findings, studies in which *L. lactis* had been administered orally have reported improvements in pig growth performance. Yu, et al. [26] reported transient improvements in ADG and feed efficiency in newly weaned pigs supplemented with *L. lactis* in feed. Similarly, in a study by Duan, et al. [27] grower-finisher pigs supplemented with *L. lactis* probiotic over 14 weeks, also demonstrated improved ADG during the grower period and improved final body weight at slaughter. The mode of administration is likely the reason for the differing outcomes, with orally administered probiotics more likely to reach the gastrointestinal tract where they can have a greater impact on growth performance. Other factors such as the frequency of administration and the dosage used may also influence the outcome of these studies. Nevertheless, the aim of this study was not to improve pig growth, rather it was to demonstrate that the nasal probiotic would not negatively affect piglet health or growth. Growth performance is considered a good indicator of pig health, and reduced health status is often associated with reduced ADG, feed intake and feed efficiency [28]. Thus, the lack of effect in this study is a positive finding.

As expected, the nasal administration of the probiotic cocktail did not negatively affect the histology of the tonsils, conchae or lungs. The minor abnormalities identified in the lungs in both groups were likely associated with the euthanasia or possibly inflammation or pneumonia caused by environmental factors within the pig unit. Indeed, euthanasia with sodium pentobartitone has been associated with congestion and oedema of the lungs [29,30,31]. The occurrence of neutrophils, thickening of alveolar septae and interlobular oedema are indicative of inflammation and possibly pneumonia [32]. Respiratory diseases such as pneumonia can be caused by several pathogens as well as other environmental factors such as inappropriate ammonia and dust levels, temperatures and relative humidity [33]; such factors may have contributed to these findings in the current study. While the current probiotic did not significantly impact lung histology in this study, Chen, et al. [34] previously showed that intranasal administration of *Lactobacillus johnsonii* improved lung alveolarization and angiogenesis in new-born mice reared in hypoxia conditions seven days after birth. The differing outcomes may be related to the differing animal species, probiotic strains, the duration of treatment and the health status of the animals used [34]. However, further research is required to elucidate the potential effects of intranasal probiotics on lung histopathology in pigs.

While the bacterial cocktail did not influence pig growth performance or histopathology, it did effect gene expression in the nasal conchae. The nasal conchae were selected for gene expression analysis as the conchae are directly exposed to the bacterial cocktail upon administration. The expression of porcine beta-defensin 2 (*pBD2*) was higher in the bacterial cocktail group compared to the placebo group at 24 h and 14 d post administration. The expression of the cytokine *IL1B* was reduced at 24 h post administration and increased at 96 h post administration in the bacterial cocktail group compared with the placebo group. Porcine beta-defensin 2 is an antimicrobial peptide which has strong antibacterial activity against both gram-positive and gram-negative bacteria including multi-resistant bacteria [35,36]. IL-1β is a potent pro-inflammatory cytokine that is crucial for host-defence responses to infection and injury [37]. It is released by monocytes, macrophages and non-immune cells in response to cell injury, infection, invasion and inflammation [38]. Previously, oral administration of pBD2 to pigs challenged with *Escherichia coli* was associated with a reduced inflammatory response, including a reduction in *IL1β* expression in the mucosa of the jejunum and improved intestinal morphology [39]. This observation is like the trend observed in the current study at 24 h, whereby the expression of *IL1B* is reduced, while *pBD2* expression is increased. These findings suggest that elevated pBD2 may help control the damage associated with inflammatory responses by regulating the expression of pro-inflammatory cytokines. Interestingly, the expression of *pBD2* was not increased at 96 h post administration and this may help to explain the increased expression of *IL1β* at this time point. On the other hand, nasal administration of the probiotic *Lactobacillus rhamnosus GG* to mice challenged with influenza virus, was previously associated with an increase in *IL1β* and other inflammatory markers [40], suggesting that the type of immune response induced by probiotics may be influenced by the type and severity of the challenge. The expression of *TLR9* was higher in the bacterial cocktail group compared with the placebo group at 24 h post administration. Toll-like receptors recognize pathogenic components and activate immune cells to kill pathogens [41]. TLR-9 recognizes and is stimulated by unmethylated bacterial CpG-DNA [41] and can instigate cellular and humoral responses [42,43,44]. Thus, the *L. lactis* cocktail could activate immune cells through the TLR signalling, and thereby could be involved in preventing the colonisation of pathogens such as *S. aureus* [26]. Indeed, Mohamed et al. [44] demonstrated TLR9 mediated *S. aureus* killing in vitro in SAOS-2 cells, through the production of reactive oxygen species. Similar to the findings in this study, oral administration of a *L. lactis* probiotic resulted in an increase in the expression of inflammatory cytokines (IL-17, IL-18 and IL-22) in the jejunum and the TLR-2, -5 and -6 in the ileum; however, it also reduced the expression of IFNγ in the jejunum and IL-22 in the ileum at the same time [26]. Similarly, nasal application of *Bacillus subtilis* probiotic to pigs was associated with increased expression of *TLR2* and *TLR9* in the tonsils, but there were no differences in the expression of *IL1β*. Together these findings demonstrate the complexity of innate immune responses. Further investigation is needed to elucidate the immunomodulating potential of this *L. lactis* cocktail.

## 5. Conclusions

The findings of this study indicate that the *Lactococcus lactis* cocktail was well-tolerated and had no adverse effects on growth performance, health indicators, histopathology or the expression of immune markers. Some abnormalities in lung histopathology were identified; however, the commonality of the abnormalities across both treatment groups indicate that these were associated with euthanasia and/or environmental factors and were not related to the probiotic cocktail per-se. The altered expression of inflammatory cytokine, pathogen recognition receptor and antimicrobial peptide genes suggests that the probiotic cocktail has immunomodulatory potential. This immunomodulatory potential may prove beneficial in its use to reduce MRSA carriage in pigs. Further studies are required to demonstrate the MRSA reduction efficacy of the probiotic cocktail. However, it can be concluded that the cocktail is safe for intranasal application in new-born piglets.

## Figures and Tables

**Table 1:** Effect of treatment on piglet health scores from d1–5 (Least square means ± their standard errors).

Days	Treatment		*p*-Value		
	Placebo	Cocktail	Treatment	Day	Treatment × Day
1	2.08 ± 0.090	2.0 ± 0.097			0.999
2	1.79 ± 0.092	0.81 ± 0.099			<0.0001
3	1.74 ± 0.093	0.41 ± 0.099			<0.0001
4	0.65 ± 0.101	0.28 ± 0.107			0.252
5	0.65 ± 0.101	0 ± 0.108			0.0006
Overall (day 1–5)	1.38 ± 0.043	0.70 ± 0.046	<0.0001	<0.0001	

**Table 2:** Effect of bacterial cocktail on pig growth performance (least square means ± their standard errors).

	Placebo	Cocktail	*p*-Value
Preweaning			
Weaning weight (kg)	9.2 ± 0.39	8.9 ± 0.28	0.582
ADG birth to weaning (g/day)	285 ± 13.8	277 ± 10.0	0.679
Total creep intake to weaning	531 ± 81.4	659 ± 81.4	0.291
Postweaning			
Final weight (kg)	35.1 ± 3.21	34.7 ± 2.58	0.946
ADG postweaning (g/day)	560 ± 69.7	552 ± 56.1	0.946

ADG, average daily gain.

## Data Availability

The data presented in this study are available on request from the corresponding author.

## Supplementary Materials

The following supporting information can be downloaded at: https://www.mdpi.com/article/10.3390/ani13223442/s1, Table S1: Composition of the experimental diets (on an air-dry basis; kg/tonne); Table S2. Health check scoring system; Table S3: qPCR primers; Table S4: The effect of treatment on histopathology of the nasal conchae; Table S5: The effect of treatment on the histopathology of the left and right tonsil paraepiglottal; Table S6: Effect of treatment on the histopathology of the tonsil palatine; Table S7: Effect of treatment on the histopathology of the left and right tonsil retropharyngeal.
