# Supplementary material for: Safety Evaluation of an Intranasally Applied Cocktail of Lactococcus lactis Strains in Pigs
Source: Animals (Basel). 2023 Nov 8;13(22):3442. doi: 10.3390/ani13223442 (PMC10668741; doi:10.3390/ani13223442)
Supplement: Supplementary file 1 [file animals-13-03442-s001.zip › animals-2681379-supplementary.pdf]

Safety evaluation of an intra-nasally applied cocktail of *Lactococcus lactis* strains in pigs

Ruth Rattigan[1], Lukasz Wajda[1], Abel A. Vlasblom[2], Alan Wolfe[3], Aldert L. Zomer[2], Birgitta Duim[2], Jaap A. Wagenaar[2], Peadar Lawlor[1]*

[1]Pig Development Department, Animal and Grassland Research and Innovation Centre, Teagasc, Moorepark, Fermoy, Co. Cork, Ireland

[2] Faculty of Veterinary Medicine, Utrecht University, Yalelaan 1, 3584 CL, Utrecht, Netherlands

[3]School of Veterinary Medicine, University College Dublin, Belfield, Dublin 4, Ireland.

*Corresponding author: peadar.lawlor@teagasc.ie

Supplementary Table S1. Composition of the experimental diets (on an air-dry basis; kg/tonne).

**Diet Number**	**1**	**2**	**3**	**4**	**5**
**Diet type**	**Dry sow**	**Lactation**	**Starter**	**Link**	**Weaner**
**Ingredients**					
Barley	759.7	259.7	50.0	68.4	495.9
Wheat	0	455.2	0	100.0	216.8
Maize	0	0	231.0	300.0	0
Soybean meal	76.2	179.8	143.4	186.9	163.2
Full fat soybean meal	0	0	130.8	70.0	50.0
Lactoflo[1]	0	0	200.0	150.0	0
Skim milk powder	0	0	125.0	50.0	0
Soya hulls	125.3	0	0	0	0
Soya oil	14.0	66.0	85.0	38.2	40.0
Premix[2]	1.5	1.5	3.0	3.0	3.0
L-Lysine HCl	2.3	5.0	6.2	6.7	5.9
DL-Methionine	0.4	1.5	3.6	3.2	2.2
L-Threonine	1.0	2.7	3.7	3.4	2.7
L-Tryptophan	0	0.8	1.4	1.3	0.6
L-Valine	0	2.7	1.3	1.3	0.6
Limestone flour	8.5	11.5	7.0	7.5	10.5
Mono dicalcium phosphate	7.0	8.5	5.5	7.0	5.5
Salt	4.0	5.0	3.0	3.0	3.0
Phytase[3]	0.05	0.05	0.05	0.05	0.05
**Chemical composition**					
Dry matter[4]	883.0	893.0	907.0	897.0	888.0
Crude protein[4]	125.0	163.0	188.0	166.0	178.0
Ash[4]	42.0	48.0	57.0	53.0	47.0
Ether extract[4]	33.7	85.4	119.1	58.4	65.2
Crude fibre[4]	87.0	26.0	16.0	33.0	32.0
Lysine[5]	7.8	11.5	16.2	15.0	13.0
Methionine[5]	2.4	3.9	7.0	6.1	4.7
Cystine[5]	2.5	3.0	2.7	2.9	3.1
Threonine[5]	5.6	8.3	10.9	10.1	8.8
Tryptophan[5]	3.66	3.36	2.66	2.22	1.54
Digestible energy (MJ/Kg)[5]	12.51	14.86	16.20	15.00	14.27
Net energy (MJ/Kg)[5]	8.86	10.90	12.06	10.94	10.30
SID lysine[5,6]	6.6	10.7	15.3	14.1	12.0
Total calcium[5]	7.2	8.3	8.2	7.5	7.4
Digestible phosphorus[5]	3.5	3.8	4.6	4.2	3.3

[1]Lactoflo, non-hygroscopic whey permeate powder (Volac, Royston, United Kingdom)
[2]Premix provided per kilogram of complete diet Diets 1 and 2): Premix provided per kg of complete diet: Cu from copper sulphate, 15 mg; Fe from ferrous sulphate monohydrate, 70 mg; Mn from manganese oxide, 62 mg; Zn from zinc oxide, 80 mg, I from calcium iodate, 0.6 mg; Se from sodium selenite, 0.2 mg; vitamin A as retinyl acetate, 3.44 mg; vitamin D_3 as cholecalciferol, 25 mg; vitamin E as DL-alpha-tocopheryl acetate, 100 mg; vitamin K, 2 mg; vitamin B_{12}, 15 μg; riboflavin, 5 mg; nicotinic acid, 12 mg; pantothenic acid, 10 mg; choline chloride, 500 mg; Biotin, 200μg; folic acid, 5mg; vitamin B_1,2 mg; and vitamin B_6, 3 mg.Premix provided per kilogram of complete diet (Diet 3, 4 and

5): Premix provided per kilogram of complete diet: Cu from copper sulphate, 85 mg; Fe from ferrous sulphate monohydrate, 90 mg; Mn from manganese oxide, 47 mg; Zn from zinc oxide, 120 mg; I from potassium iodate, 0.6 mg; Se from sodium selenite, 0.3 mg; vitamin A as retinyl acetate, 2.1 mg; vitamin D_3 as cholecalciferol, 25 µg; vitamin E as DL-alpha-tocopheryl acetate, 100 mg; vitamin K, 4 mg; vitamin B_{12}, 15 µg; riboflavin, 2 mg; nicotinic acid, 12 mg; pantothenic acid, 10 mg; choline chloride, 250 mg; vitamin B_1, 2 mg; and vitamin B_6, 3 mg.
[3]The diet contained 500 phytase units (FYT) per kg feed (RONOZYME HiPhos GT; DSM, Belfast, UK).
[4]Analysed nutrient composition
[5]Calculated nutrient composition
[6]SID lysine = Standardized ileal digestible lysine.

Supplementary Table S2. Health check scoring system.

Category	Score			
	0	1	2	3
**Faecal score**	**NORMAL**<br>• Dry, pelleted faeces	**SOFT**<br>• Soft with shape	**MILD DIARRHOEA**<br>• Very soft or viscous liquid	**SEVERE DIARRHOEA**<br>• Watery or with blood
**Behaviour**	**NORMAL**<br>• Active	**APATHETIC**<br>• Must be stimulated to get up	**LYING**<br>• Gets up with help	**DEEP PROSTRATION**<br>• Cannot get up
**Dehydration**	**NORMAL**<br>• No signs/symptoms of dehydration	**MILD**<br>• Normal eyes<br>• Slight reduction of skin elasticity<br>• Normal capillary refill time<br>• Normal mucous membranes	**MODERATE**<br>• Slightly sunken eyes<br>• Loss of skin elasticity<br>• Normal capillary refill time<br>• Normal mucous membranes	**SEVERE**<br>• Sunken eyes<br>• Loose, wrinkled skin very evident<br>• Retarded capillary refill time<br>• Dry mucous membranes
**Clinical signs**	**NORMAL**<br>• Normal Temperature, Respiration/Cardiac rates	**MINOR**<br>• Minor changes in Temperature, Respiration/Cardiac rates	**MODERATE**<br>• Temperature ± 1°C, Respiration/Cardiac rates ±30%	**SEVERE**<br>• Temperature± 2°C, Respiration/Cardiac rates ±50%

Supplementary Table S3. qPCR primers

Gene	GenBank accession number	Primers sequences (5′ – 3′)
β-actin	U07786.1	F: CATCACCATCGGCAACGA<br>R: GCGTAGAGGTCCTTCCTGATGT
CXCL8	NM213867	F: TAGGACCAGAGCCAGGAAGA<br>R: GAACTGCAGCCTCACAGAGA
IL6	M86722.1	F: TGGATAAGCTGCAGTCACAG<br>R: ATTATCCGAATGGCCCTCAG
IL1β	M86725.1	F: AAGTGATGGCTAACTACGGTGAC<br>R: ATCTGCCTGATGCTCTTGTTCC
TNFα	X57321.1	F: CACCACGCTCTTCTGCCTACTGC<br>R: TCGGCTTTGACATTGGCTACAA
pBD2	AY506573.1	F: ACCTGCTTACGGGTCTTG<br>R: CTCTGCTGTGGCTTCTGG
TLR2	AB072190	F: ACATGAAGATGATGTGGGCC<br>R: TAGGAGTCCTGCTCACTGTA
TLR9	AB071394	F: GTGGAACTGTTTTGGCATC<br>R: CACAGCACTCTGAGCTTTGT

β -actin, beta-actin; CXCL8, C-X-C motif chemokine ligand 8 (IL8); IL6, interleukin 6; IL1 β, interleukin 1-beta; TNFα, tumour necrosis factor alpha; pBD2, beta-defensin 2; TLR2, toll like receptor 2; TLR9, toll like receptor 9.

Supplementary Table S4. The effect of treatment on histopathology of the nasal conchae.

Treatment Group	Placebo	Bacterial Cocktail
	**No abnormalities**	
24h after birth	3/6	6/6
96h after birth	6/6	6/6
14d after birth	6/6	6/6
	**Non-significant findings**	
24h after birth	3/6	0/6
96h after birth	0/6	0/6
14d after birth	0/6	0/6

*** Minor changes in epithelium and/or cellular debris in the lumen**

Supplementary Table S5. The effect of treatment on the histopathology of the left and right tonsil paraepiglottal.

	Left		Right	
**Treatment Group**	**Placebo**	**Bacterial Cocktail**	**Placebo**	**Bacterial Cocktail**
		**No lymphoid tissue collected**		
24h after birth	6/6	5/6	5/6	6/6
96h after birth	5/6	6/6	6/6	6/6
14d after birth	4/6	4/6	4/6	3/6
		**No abnormalities**		
24h after birth	0/6	1/6	1/6	0/6
96h after birth	0/6	0/6	0/6	0/6
14d after birth	2/6	2/6	2/6	3/6
		**Non-significant findings**		
24h after birth	0/6	0/6	0/6	0/6
96h after birth	1/6	0/6	0/6	0/6
14d after birth	0/6	0/6	0/6	0/6

*** Normal findings of neutrophils and eosinophils scattered throughout tissue.**

Supplementary Table S6. Effect of treatment on the histopathology of the tonsil palatine.

Treatment Group	Placebo	Bacterial Cocktail
	**No lymphoid tissue collected**	
24h after birth	0/6	3/6
96h after birth	0/6	0/6
14d after birth	0/6	0/6
	**No abnormalities**	
24h after birth	3/6	0/6
96h after birth	0/6	0/6
14d after birth	1/6	0/6
	***Non-significant findings**	
24h after birth	3/6	3/6
96h after birth	6/6	6/6
14d after birth	5/6	6/6

*** Normal findings of neutrophils and eosinophils scattered throughout tissue and/or cellular debris and degenerate inflammatory cells in the crypts.**

Supplementary Table S7. Effect of treatment on the histopathology of the left and right tonsil retropharyngeal.

	Left		Right	
**Treatment Group**	**Placebo**	**Bacterial Cocktail**	**Placebo**	**Bacterial Cocktail**
**No lymphoid tissue collected**				
24h after birth	4/6	1/6	2/6	4/6
96h after birth	3/6	3/6	1/6	4/6
14d after birth	3/6	2/6	2/6	1/6
**No abnormalities**				
24h after birth	1/6	5/6	4/6	2/6
96h after birth	1/6	1/6	1/6	1/6
14d after birth	2/6	4/6	0/6	2/6
***Non-significant findings**				
24h after birth	1/6	0/6	0/6	0/6
96h after birth	2/6	2/6	4/6	1/6
14d after birth	1/6	0/6	4/6	3/6

***Normal findings of neutrophils and eosinophils scattered throughout tissue and/or cellular debris and degenerate inflammatory cells in the crypts.**
